# Isolation of arsenic accumulating bacteria from garbage leachates for possible application in bioremediation

**Published:** 2019-02

**Authors:** Mojtaba Taran, Roohollah Fateh, Shima Rezaei, Mohammad Khalifeh Gholi

**Affiliations:** 1 Department of Biology, Faculty of Sciences, Razi University, Kermanshah, Iran; 2 Cellular and Molecular Research Center, Qom University of Medical Sciences, Qom, Iran; 3 Department of Microbiology and Immunology, Faculty of Medicine, Qom University of Medical Sciences, Qom, Iran; 4 Research Center for Environmental Pollutants, Qom University of Medical Sciences, Qom, Iran

**Keywords:** *Bacillus* sp., Bioremediation, Arsenic, Taguchi method, Garbage leachate

## Abstract

**Background and Objectives:**

Bioremediation is a process to reduce toxic heavy-metals, such as arsenic, in the environment using microorganisms. This study aimed to isolate arsenic remediating microbial strains from garbage leachates and to evaluate the effects of several factors on bioremediation by isolated strains.

**Materials and Methods:**

After isolating arsenic-resistant bacteria from garbage leachates and determining their MIC values, Taguchi design of experiments was used to evaluate the effect of arsenic concentration, pH solution, temperature, and contact time on arsenic bioremediation by isolated bacteria.

**Results:**

The results revealed that 3 arsenic-resistant strains of genus *Bacillus* characterized as KL1, KL4, and KL6 had arsenic bioremediation activity. Based on the results, the highest bioremediation of arsenic by *Bacillus* sp. KL1 was obtained as 77% after 24 hours at 40°C, pH 5, and 150 ppm concentration. However, the maximum bioremediation of arsenic by KL4 (91.66%) and KL6 (88%) was achieved after 24 hours at 40°C, pH 5, and 60 ppm concentration and at 35°C, 90 ppm concentration, pH 5 after 36 hours, respectively.

**Conclusion:**

The results presented here may facilitate improvements in the eliminating arsenic from contaminated sites and reducing environmental pollutions.

## INTRODUCTION

Arsenic (As) is a toxic metalloid released into the environment either by natural phenomena (weathering, volcanic activity) or by anthropogenic activities such as mining, smelting, and combustion of fossil fuels (1, 2). Several oxidation states of arsenic include+5 (arsenate), +3 (arsenite), 0 (elemental arsenic), and −3 (arsine), which are found in the nature. However, the most common environmental oxidation states of arsenic are the pentavalent As (V) and trivalent As (III) forms. As (III) is more toxic than others (3, 4).

Chronic arsenic poisoning in the general population has been widely reported in many regions of the world (4). Thus, elevated arsenic levels have been reported in soils and ground water worldwide. In drinking water, the maximum arsenic concentration limit recommended by World Health Organization (WHO) is 0.01 mg/L (4, 5). High levels of arsenic in drinking water can affect human health and have immediate toxic effects on them (5). Many clinical presentations have been reported from chronic arsenic toxicity, including skin lesions (eg, hyperkeratosis, hyperpigmentation, desquamation and hair loss), cancer of various organs, such as skin, bladder, kidney, and lung, high blood pressure, diseases of the legs and feet, blood vessels, and reproductive disorders (6).

Arsenic is generally toxic to life, however, some bacteria are resistant to arsenic and can use arsenic compounds as electron donors, electron acceptors, or possess arsenic detoxification mechanisms (2, 3). The most widespread resistance mechanisms detected in bacteria are *ars* operons, which are either chromosomally or plasmid encoded. The most common types of these operons contain 5 (*ars*RDABC) or 3 (*ars*RBC) genes (2).

Development of effective tools and techniques to manage environmental pollutions is an interesting research field in biotechnology (7). Bioremediation is the process for reduction of environmental pollutants using microorganisms. Bioremediation of several forms of arsenic by microbial community involves oxidation, reduction, and methylation and intracellular bioaccumulation of these compounds (8, 9).

Waste leachate production is one of the biggest problems in the world because liquid wastes contaminate surrounding soil surfaces and ground water. Garbage leachate contaminated soils are good sources to isolate such kind of bioremediation agents having potential to degrade the waste compounds (7).

This study aimed to identify the bacteria in the municipal garbage leachates that have bioremediation activity for arsenic and to evaluate optimization conditions using Taguchi method to have the highest bioremediation activity.

## MATERIALS AND METHODS

### Samples collection.

A total of 50 samples of waste leachate were collected in labeled sterile bottles from the main municipal solid waste leachate contaminated site in Kermanshah province, Iran, and transferred to the laboratory. All samples were kept at 4°C for further experiments.

### Isolation of arsenic-resistant bacteria.

Bacteria from the waste leachates were isolated using a serial dilution procedure and up to 10^−5^ dilutions were prepared. To isolate arsenic-resistant bacteria, 100 μL of each suspension was spread on arsenite-containing (0.4 mM) nutrient agar medium. The plates were incubated at 35°C for 72 hours, then, the presence of bacterial colonies on plates was investigated (10).

To purify arsenic-resistant bacteria, single emerged colony on plates were picked up and streaked on arsenite-containing (0.4 mM) nutrient agar media. Finally, automated 16SrDNA gene sequencing and biochemical tests (oxidase, catalase, urease, indole production, nitrate reduction, MR, VP, citrate utilization) were used to characterize the bacteria which were grown on the arsenic-containing media. Gene sequencing and biochemical analysis were performed at Iranian Biological Resource Center.

### Detection of the minimum inhibitory concentration of arsenic.

The minimum inhibitory concentration (MIC) was assessed using a preparation of serial dilutions of arsenic (25, 50, 75, 100, 125, 150, 175, 200, 225, 250, 275, 300 ppm). Also, 100 microliters of each dilution of arsenic was added to the surface of a nutrient agar plate. The bacterial suspension was prepared and adjusted spectrophotometrically at 625 nm (OD625) to match a turbidity of 1.5 × 10^8^ CFU/mL (equivalent to 0.5 McFarland standards). Then, 20 μL of 0.5 McFarl and of arsenic-resistant bacterial suspension was streaked on the surface of the agar plates. These plates were then incubated at 35°C for 7 days (7, 11). The plates were assessed daily for bacterial growth against control to ensure reliable results. Moreover, 3 different colonies of bacterial isolates with highest MIC were selected and identified by the Iranian Biological Resource Center, according to 16S rDNA gene analysis.

### Design of experiments (DOEs).

Experiments were designed by Taguchi statistical method to evaluate the effects of arsenic concentration, pH, temperature, and contact time on bacterial bioremediation of arsenic (Table 1).

**Table 1.** Factors and their levels utilized in the Taguchi DOEs to evaluate bioremediation of arsenic

**Factor**	**Level 1**	**Level 2**	**Level 3**
Time (h)	12	24	36
pH	5	7	9
Temperature (°C)	30	35	40
Concentration (ppm)^a^	30	60	90
Concentration (ppm)^b^	75	150	225

a Concentrations used for KL4 and KL6 strains

b Concentrations used for KL1 strain

### Bioremediation of arsenic according to DOEs.

A single colony of bacteria with the highest MIC was cultured in 100 mL nutrient broth and incubated at 37°C and 120 rpm. The arsenic working solutions were prepared according to DOEs. Then, 5 mL of each arsenic stock solution was mixed with 5 mL of bacterial suspension to prepare working concentrations designed by Taguchi method. Then, the pH of suspensions was adjusted with sodium hydroxide or hydrochloric acid (0.1 M) according to DOEs. All samples were incubated at certain temperatures and times for each experiment. After incubation, the samples were centrifuged at 12000 rpm for 5 minutets and supernatants were collected. Atomic absorption spectrophotometry (GBC-q02) was used to measure arsenic concentrations in these solutions (7). The experiments were performed in triplicate to ensure their reproducibility.

### Statistical Analysis.

The data obtained from Taguchi experiments was analyzed using the fixed-effects model of analysis of variance (ANOVA) in Qualitek-4 software (V. 14.5, Nutek Inc., MI, USA).

## RESULTS

### Isolation of arsenic-resistant bacteria lcolonies.

Different colonies were grown on the nutrient agar plates supplemented with 0.4 mM arsenite. Severalcolonies were selected and purified for further studies to check their resistance to arsenic. Three bacterial species were isolated as arsenic-resistant strains based on the biochemical and 16S rDNA gene analysis. The bacteria belonged to genus *Bacillus* and were characterized as *Bacillus* sp. KL1, KL4 and KL6 (Table 2).

**Table 2.** Biochemical charaterization of isolated bacteria

**Bacterial isolate**	**Oxidase**	**Catalase**	**Nitrate reduction**	**Urease test**	**Citrate utilization**	**Methyl Red**	**Voges-Prauskauer**	**Indole**
*Bacillus* sp. KL1			+	+		+		+
*Bacillus* sp. KL4			+	+		+		+
*Bacillus* sp. KL6			+	+		+		+

### Determining arsenic MIC.

The MIC of arsenic was determined for these species. Based on MIC values, the maximum arsenic tolerance for KL1 was obtained to be 225 μg/mL and 90 μg/mL for both KL4 and KL6 species.

### Different levels inarsenic bioremediation.

The results of the Taguchi optimization method showed 9 orthogonal arrays of experiments. These results indicated that the highest bioremediation of arsenic by *Bacillus* sp. KL1 (77%) was obtained at 4^th^ run (40°C, 150 ppm, pH 5 and 24 h). However, the highest bioremediation of arsenic by KL4 (91.66%) and KL6 (88%) was at 4^th^ (40°C, 60 ppm, pH 5 and 24 h) and 7th (35°C, 90 ppm, pH 5 and 36 h) runs, respectively (Table 3).

**Table 3.** Taguchi DOEs and corresponding bioremediation of arsenic by *Bacillus* sp. KL1

**Experiment No**	**Time (h)**	**pH**	**Concentration (ppm)**	**Temperature (°C)**	**mediation rate (%) Biore**
1	12	5	75	30	75.77
2	12	7	150	35	73.11
3	12	9	225	40	39
4	24	5	150	40	77
5	24	7	225	30	60.44
6	24	9	75	35	42.11
7	36	5	225	35	73
8	36	7	75	40	40
9	36	9	150	30	22

The rate of arsenic bioremediation by the 3 strains of *Bacillus* was measured at several levels of different factors (time, pH, concentration, and temperature).

Maximum bioremediation of arsenic by *Bacillus* sp. KL1 (62.62, 75.25, 57.47 and 62.74 %) was observed with different parameters at levels 1 (12 h; pH 5), level 3 (225 ppm), and at level 1 (35°C). In *Bacillus* sp. KL4, maximum arsenic bioremediation (87.62, 89.80, 88.95, 87.66%) was obtained at levels 1 (12 h; pH 5), 2 (60 ppm) and 1 (30°C). However, maximum bioremediation by *Bacillus* sp. KL6 (78.22, 84.37, 76.33 and 79.92%) was observed at levels 1 (12 h; pH 5), 3 (90 ppm) and 2 (35°C) (Table 4).

**Table 4.** The effects of different levels of factors on arsenic bioremediation by *Bacillus* sp. KL1, KL4 and KL6

**Factors**	**Level 1 (%)**			**Level 2 (%)**			**Level 3 (%)**		

	**KL1**	**KL4**	**KL6**	**KL1**	**KL4**	**KL6**	**KL1**	**KL4**	**KL6**
Time (h)	62.62	87.62	78.22	59.85	87.58	74.40	45	86.13	70.33
pH	75.25	89.80	84.37	57.85	86.61	70.84	34.36	84.92	67.73
Concentration (ppm)	52.62	86.24	71.11	57.36	88.95	75.51	57.47	86.14	76.33
Temperature (°C)	52.73	87.66	72.37	62.74	87.02	79.92	52	86.65	70.66

The effects of interacting factor pairs on the arsenic bioremediation activity demonstrated that the interactions of concentration and temperature (77.06), concentration and time (54.43), and concentration and pH (56.14) were the most effective pair on arsenic bioremediation of *Bacillus* sp. KL1, KL4, and KL6, respectively (Table 5). On the other hand, it was indicated that interacting factor pair time and pH (12.63) has the minimum effect on the bioremediation activity of KL1 strain, whereas, time and temperature showed the minimum effect on the arsenic bioremediation by KL4 and KL6 strains (7.18 and 1.39 respectively).

**Table 5.** Estimating the effects of intracting factors pairs on arsenic bioremediation by *Bacillus* sp. KL1, KL4 and KL6

***Bacillus* sp. KL1**		

Intracting factor pairs	Levels	Contribution (%)
Time × Temperature	12 h × 35°C	14.24
pH × Temperature	5 × 35°C	14.03
pH × Concentration	5 × 225 ppm	28.98
Time × pH	12 h × 5	12.63
Concentration × Temperature	225 ppm × 35°C	77.06
Time × Concentration	12 h × 225 ppm	34.13

***Bacillus* sp. KL4**		

Intracting factor pairs	Levels	Contribution (%)
Time × Temperature	12 h × 30°C	7.18
pH × Temperature	5 × 30°C	29.04
pH × Concentration	5 × 60 ppm	19.05
Time × pH	12 h × 5	25.32
Concentration × Temperature	60 ppm × 30°C	52.93
Time × Concentration	12 h × 60 ppm	54.43

***Bacillus* sp. KL6**		

Intracting factor pairs	Levels	Contribution (%)
Time × Temperature	12 h × 35°C	1.39
pH × Temperature	5 × 35°C	10.01
pH × Concentration	5 × 90 ppm	56.14
Time × pH	12 h × 5	16.85
Concentration × Temperature	90 ppm × 35°C	23.82
Time × Concentration	12 h × 90 ppm	40.46

The ANOVA results indicated that pH parameter with the maximum sum of squares (S), variance (V), and percentage influence in KL1 (2526.02, 1263.01, and 75.92, respectively), KL4 (36.955, 18.477 and 63.57), and KL6 (469.018, 234.509 and 62.1) strains is the most influential factor on arsenic bioremediation (Table 6).

**Table 6.** ANOVA test for Taguchi results

**Factor**	**DOF (f)**			**Sum of Sqrs (S)**			**Variance**			**Pure sum (S')**			**Percent (%)**		

	**KL1**	**KL4**	**KL6**	**KL1**	**KL4**	**KL6**	**KL1**	**KL4**	**KL6**	**KL1**	**KL4**	**KL6**	**KL1**	**KL4**	**KL6**
Time (h)	2			538.93	4.349	93.335	269.46	2.174	46.667	538.93	4.349	93.335	16.19	7.48	12.356
pH	2			2526.02	36.955	469.018	1263.01	18.477	234.509	2526.02	36.955	469.018	75.92	63.567	62.091
Concentration (ppm)	2			46.066	12.266	47.371	23.033	7.633	23.685	46.066	12.266	47.371	1.38	26.26	6.271
Temperature (°C)	2			215.95	1.564	145.638	107.97	0.782	72.819	215.95	1.564	145.638	4.69	2.69	19.28

It was found that the concentration parameter hadthe lowest effect on arsenic bioremediation by KL1 (1.38) and KL6 (6.271) strains, but time had the lowest effect on bioremediationin KL4 strain (2.69).

## DISCUSSION

Nowadays, the use of microorganisms in biological removal of toxic compounds, such as arsenic, is potentially important (4). The first step in identifying bacteria with the ability of bioremediation is separation of resistant bacteria to high concentrations of heavy metals (12). There are many arsenic-resistant bacteria isolated from arsenic-rich environments. Ghodsi, in southwest of Isfahan, Iran, isolated 3 arsenic-resistant bacteria related to *Bacillus* and *Corynebacterium* genus, whose maximum MIC was 128 mM/l (12). Sivakumar Selvi, in Tamilnadu, South India, isolated 2 arsenic-resistant bacteria from agricultural soils that belonged to the genera *Enterobacter asburiae* and *Enterobacter cloacae*. The MICs of both isolates were 40 mM and 400 mM for sodium arsenite and sodium arsenate (11). In this study, 3 bacterial strains of genus *Bacillus* were isolated from garbage leachates that grew in the presence of elevated arsenic concentrations. The finding of the present study revealed that arsenic-resistant bacteria displayed high levels of resistance to feasible bioremediation strategies. Among isolated strains, *Bacillus* sp. KL1, with the highest level of tolerance to toxic effects of arsenic (MIC=225 μg/mL), can be primarily considered as an appropriate candidate to resolve arsenic environmental pollution. However, the bioremediation activity of bacteria is greatly affected by several factors (concentration of toxic compounds, pH, contact time, temperature) and optimization of the conditions could result in high biosorption and bioremediation of heavy metals (4).

Taguchi designs prepare a potent and impressive method to design processes that operate constantly and optimally over a variety of conditions (13). However, in the present study, Taguchi optimization method was used to determine optimized conditions for bioremediation of arsenic by isolated strains. The results of the Taguchi optimization method represented 9 orthogonal arrays of testing (Table 4); the optimum conditions for the highest bioremediation by *Bacillus* sp. KL1, KL4 were achieved at the 4^th^ run and at the 7^th^ run for KL6 strain.

In the present study, the effects of parameters, including contact time, solution pH, arsenic concentration, and temperature, were assessed on bioremediation activity of *Bacillus* sp. KL1, KL4 and KL6. Contact time is an important parameter to achieve the maximum biosorption (14). The results of this study indicated that the highest uptake of arsenic by *Bacillus* sp. KL1, KL4 and KL6 takes a shorter time (12 h) and metal biosorption reduces with increasing contact time. This is perhaps due to the induction of metal exudation into solution by bacteria (15).

The present finding also supports previous studies on other bacteria (10, 16–18), which concluded that efficient bioremediation of arsenic occurs in the solution pH, range 5–7. This study indicated that pH 5 is optimum for the maximum biosorption of arsenic by 3 isolated *Bacillus* strains. Among the plausible explanation for this result is that metal biosorption depends on the protonation or deprotonation of the cell wall functional groups (19). At low pH, by creating a positive charge on the metal binding site due to the high concentration of proton, metal cations and protons compete for binding sites, which results in lower uptake of metal (19). On the other hand, at values of pH higher than the optimum, formation of hydroxylated complexes of the metal cations will compete with binding site, resulting in decreasing metal biosorption (19–21). However, maximum biosorption of metal cations has been reported in weak acidic and neutral pH due to a more negative charge of the bacterial cell surface (10, 16–18).

The results obtained from this study revealed that optimum temperature for biosorption by the isolated strain ranged 30°C–35°C, as the growth of the selected strain increases in this temperature range. Consistent with findings by Sari et al. (14), it was found that the bioremediation activity of all 3 strains was reduced at high temperature (40°C), as the raising temperature destroys the surface metal binding site in the bacterial cell wall (7).

The effect of initial arsenic concentration on the biosorption capacity of *Bacillus* sp. KL1, KL4, and KL6 was studied under optimum conditions. The results provided evidence that the maximum percentage removal of arsenic by *Bacillus* sp. KL1 was observed when the initial arsenic concentration varied from 30 ppm to 60 ppm (88.95%), but the arsenic biosorption decreased with increasing initial concentration to 90 ppm. These results may be described by an increase in the number of metal ions and the lack of free binding sites on the biosorbent cell wall at higher concentration levels. Never the less, at a low concentration, there are numerous free binding sites on the bacterial surface and, hence, metal biosorption is highly effective.

In 2 strains, KL1 and KL6, the biosorption of arsenic increased with raising the initial concentration of metal ions. It is likely that the number of available metal uptake sites on these bacterial surfaces is more than KL1 strain. Therefore, reduction of the metal uptake may occur at the concentrations higher than this experiment.

The results revealed that the effect of one factor on arsenic bioremediation activity is dependent on its interaction with other factors. Arsenic concentration at the optimum level shows relatively less effect than other factors on arsenic bioremediation activity of the studied strains, but the maximum bioremediation activity of KL1, KL4, and KL6 was achieved in its interaction with the optimal level of temperature, time and pH, respectively.

The present study was designed to isolate several bacteria with arsenic biosorption activity from garbage leachates and determine the effect of different factors on arsenic bioremediation of these bacteria. One of the significant findings of this study was that 3 strains of the genus *Bacillus* were isolated from garbage leachates with high resistance to arsenic which can be considered for biosorption. It was also shown that Taguchi optimization approach is an effective tool for modeling and analyzing influential factors for the maximum arsenic bioremediation by these strains. The second major finding was that the optimum bioremediation of arsenic was influenced by 4 main factors: contact time, arsenic concentration, solution pH, and temperature.

The results of this investigation showed that despite the high resistance of KL1 strain to arsenic, the highest biosorption was obtained by KL4 strain (91.66%) and then by KL6 strain (88%) in optimum conditions. In general, the most important results of this study was the effectiveness of 3 *Bacillus* sp. KL1, KL4, and KL6 for bioremediation of arsenic, and it looks promising that these microorganisms may be applied to treat environmental pollutions.
